# Hormetic Property of Ginseng Steroids on Anti-Oxidant Status against Exercise Challenge in Rat Skeletal Muscle

**DOI:** 10.3390/antiox6020036

**Published:** 2017-05-19

**Authors:** Ming-Fen Hsu, Szu-Hsien Yu, Mallikarjuna Korivi, Wei-Horng Jean, Shin-Da Lee, Chih-Yang Huang, Yi-Hung Liao, Jessica Lu, Chia-Hua Kuo

**Affiliations:** 1 Graduate Institute of Sports Training, University of Taipei, 11153 Taipei, Taiwan; mingfenhsu@gmail.com; 2 Department of Leisure Industry and Health Promotion, National Ilan University, 26047 Ilan, Taiwan; shyu0918@gmail.com; 3 Department of Sports Sciences, University of Taipei, 11153 Taipei, Taiwan; mallik.k5@gmail.com; 4 Department of Anesthesiology, Far Eastern Memorial Hospital, 22060 New Taipei, Taiwan; dtpc25@gmail.com; 5 Graduate Institute of Physical Therapy and Rehabilitation Science, China Medical University, 40402 Taichung, Taiwan; shinda@mail.cmu.edu.tw; 6 Department of Healthcare Administration, Asia University, 41354 Taichung, Taiwan; 7 Institute of Basic Medical Science, China Medical University, 40402 Taichung, Taiwan; cyhuang@mail.cmu.edu.tw; 8 Department of Exercise and Health Science, National Taipei University of Nursing and Health Sciences, 11219 Taipei, Taiwan; yihungliao.henry@gmail.com; 9 Pegasus Pharmaceuticals Group Inc. Richmond, V6X 1Z7 BC, Canada; miniolu@gmail.com

**Keywords:** adaptogen, anti-oxidant enzyme, ginsenosides, MDA, ROS, dammarane sapogenins

## Abstract

Background: Existing literature on anti-oxidant capacity of ginseng has been inconsistent due to variance in the profile of ginseng steroids (Ginsenosides) that is because of differences in seasons and species. Methods: We used various doses of ginseng steroids to determine its effect on oxidative stress and anti-oxidant capacity of rat skeletal muscle against exercise. Results: Under non-exercise conditions, we found increased thiobarbituric acid reactive substance (TBARS) levels and decreased reduced/oxidized glutathione ratio (GSH/GSSG) in rat skeletal muscle as dose increases (*p* < 0.05), which indicates the pro-oxidant property of ginseng steroids at baseline. Intriguingly, exhaustive exercise-induced increased TBARS and decreased GSH/GSSG ratio were attenuated with low and medium doses of ginseng steroids (20 and 40 mg per kg), but not with high dose (120 mg per kg). At rest, anti-oxidant enzyme activities, including catalase (CAT), glutathione reductase (GR) and glutathione S-transferase (GST) were increased above vehicle-treated level, but not with the high dose, suggesting a hormetic dose-response of ginseng steroids. Conclusion: The results of this study provide an explanation for the inconsistent findings on anti-oxidative property among previous ginseng studies. For optimizing the anti-oxidant outcome, ginseng supplementation at high dose should be avoided.

## 1. Introduction

An acute bout of exhaustive exercise has been shown to increase lipid peroxidation and decrease mitochondria function in exercised muscle [1,2], which is associated with excessive production of reactive oxygen species (ROS). Citrate synthase (CS) activity in mitochondria of skeletal muscle decreases immediately after exercise [3]. In skeletal muscle, enzymatic anti-oxidants, such as superoxide dismutase (SOD), CAT, glutathione peroxidase (GPx), GR and GST, and non-enzymatic anti-oxidative compounds, such as GSH [4] are able to scavenge the ROS and protect cells from overwhelming oxidative damage [5,6]. Supplementation of antioxidants has been shown to prevent exhaustive exercise-induced mitochondrial damage, possibly through maintaining the redox homeostasis [7]. On the other hand, chronic exercise training reported to induce antioxidant enzymes and offer protection from oxidative damage [5]. 

Ginseng has been claimed to increase the anti-oxidant capability against oxidative stress [8,9]. However, previous ginseng studies present miscellaneous results on anti-oxidant capacity [10]. It has been shown that metabolic action of ginseng could be changed in accordance with the ginsenoside profile at different seasons [11]. Cultivation methods, soil composition and species type are also responsible for the inconsistent profile of ginseng steroids [12,13]. 

To the best of our knowledge, the effect of ginseng steroid supplementation on lipid peroxidation and anti-oxidant status of skeletal muscle induced by exhaustive exercise has not been documented in vivo. Dammarane ginsenoside is the major class of steroids enriched in ginseng, which contains protopanaxadiol (PPD), protopanaxatriol (PPT), Rg3, Rh1, and Rh2. In this study, for the first time we evaluated the in vivo dose response of the ginseng steroids on oxidative stress and anti-oxidant capacity of non-exercised and exercise-challenged rat skeletal muscles. We hypothesized that ginseng steroids pretreatment may attenuates exhaustive exercise-induced oxidative stress in the skeletal muscle.

## 2. Materials and Methods 

### 2.1. Animals

Eighty male Sprague Dawley rats (410 ± 10 g, aged ~4 months old) purchased from LASCO Corporation (Taipei, Taiwan) were housed in a cage with standard laboratory chow (PMI Nutrition International, Brentwood, MO, USA) and tap water *ad libitum*. All animals were kept in an animal room with 12/12 h light/dark cycle, 22 ± 2 °C and 50% relative humidity in Animal Center at the University of Taipei for one week before experiment. All animal experiment protocols were approved by the Ethic Committee of University of Taipei (Taipei, Taiwan) (Approval No. 9807001), in accordance with the Guide for the Care and Use of Laboratory Animals and Taiwan’s Animal Protection Law, 1998.

### 2.2. Ginseng Steroids

**Figure 1:**
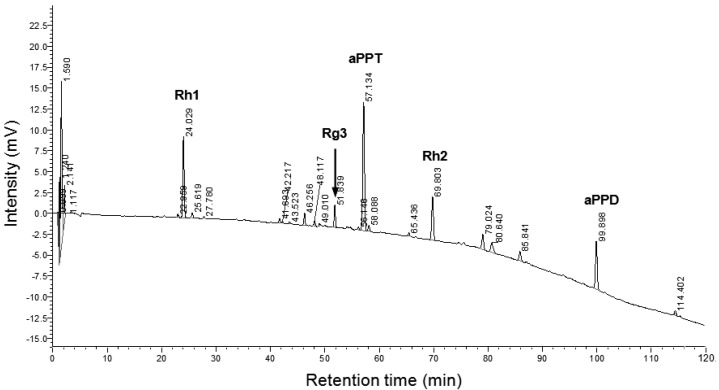
HPLC profile of ginseng steroids. The main compound contains Rh1 (17.19%), Rg3 (3.96%), Rh2 (9.56%), 20(S)-aglycone protopanaxadiol (aPPD) (7.02%) and 20(S)-aglycone protopanaxatriol (aPPT) (20.51%).

The ginseng steroids used in the study were the dammarane sapogenins (Panagin DS-1227, Pegasus Pharmaceuticals Group Inc. Richmond, BC, Canada), which were prepared by removal of long glycosyl side chain to facilitate absorption gastrointestinal [14]. Ginseng steroids (Ginsenosides) profile of HPLC analysis is shown in Figure 1. The ginseng steroids used in the study contains Rh1 (C_36_H_64_O_9_) (17.19%), Rg3 (C_42_H_72_O_13_) (3.96%), Rh2 (C_36_H_64_O_8_) (9.56%), 20(S)-aglycone protopanaxadiol (aPPD) (C_30_H_54_O_3_) (7.02%) and 20(S)-aglycone protopanaxatriol (aPPT) (C_30_H_54_O_4_) (20.51%).

### 2.3. Experimental Design

Animals were first randomly assigned into the following 4 main groups (N = 20 for each group): Vehicle, G20 (20 mg/kg body weight of ginseng steroids), G60 (60 mg/kg body weight of ginseng steroids) and G120 (120 mg/kg body weight of ginseng steroids) after seven days of housing familiarization. Experimental animals received ginseng steroid solution (Dissolved in 0.9% saline) daily at the dose of 20, 60, 120 mg/kg body weight by oral intubation using gastric gavage for 10 weeks. The same volume of 0.9% saline was given to the vehicle-treated group. Ginseng steroids was dissolved and diluted in 0.9% saline. Depend on rat’s body weight, approximatively 1 ml of saline or ginseng steroid solution was orally delivered. After the 10-week supplementation, animals in each of 4 main groups were equally divided into non-exercise and exhaustive swimming exercise subgroups. Numbers of animals in each group are indicated in Table 1.

### 2.4. Exercise Protocol

Animals in the exercise group were subjected to an acute exhaustive swimming exercise at 33 ± 1 °C. For familiarization, rats were swum in water pool for 10 min daily three days before the exercise challenge. On the day of exercise performance, rats were placed on water surface with lead ingot hanged to tail at 3% body weight and allowed to swim until exhaustion. Rats were anesthetized immediately after exhaustion together with their non-exercise vehicle-treated group for tissues collection.

### 2.5. Tissue Isolation

All rats were sacrificed under anesthesia with an intraperitoneal injection of choral hydrate (400 mg/kg body weight). The tibialis anterior (TA) muscles were excised and frozen immediately in liquid nitrogen, and then stored at −80 °C until analyses. TA consists of primarily of fast twitch muscle fibers. Muscle sample (Approximately 100 mg) was homogenized at 1:10 ratio (W/V) in solution containing 50 mM Tris base, pH 7.5. Homogenates were centrifuged at 10,000 *g* for 10 min at 4 °C and the supernatant was collected for the estimation of TBARS and reduced/oxidized glutathione levels, and enzyme activities of SOD, CAT, GPx, GR, GST and XO.

### 2.6. Lipid Peroxidation

The lipid peroxidation marker, TBARS, was measured according to the method described by Ohkawa et al. [15]. Muscle tissue was homogenized in phosphate buffer (50 mM, pH 7.0) and then centrifuged at 10,000 *g* for 10 min at 4 °C. Thiobarbituric acid (TBA) was added to supernatant to generate chromophoric MDA-TBA compound under high temperature (90–100 °C). The MDA-TBA level was assessed spectrophotometrically at 450 nm. The TBARS levels are standardized by protein content of each sample. Total protein content was determined by Bio-Rad Protein Assay reagent (BioRad Laboratories, Hercules, CA, USA).

### 2.7. Reduced and Oxidized Glutathione

The glutathione assay kit (Cayman Chemical Company, Ann Arbor, MI, USA) was used to detect the levels of GSH and GSSG. The principle is based on the reaction between GSH and DTNB (5,5’-dithio-*bis*-2-nitrobenzoic acid) to form a colored product TNB (5-thio-2-nitrobenzoic acid). The concentration of TNB was measured spectrophotometrically at 405 nm by enzyme-linked immunosorbent assay (ELISA) reader (Tecan GENios, A-5082, Tecan, Salzburg, Austria).

### 2.8. Anti-Oxidant Enzyme

Anti-oxidant enzyme activity was determined using commercial kits (Cayman Chemical Company, Ann Arbor, MI, USA). For SOD activity assay, muscle samples were homogenized in 4-(2-Hydroxyethyl)piperazine-1-ethanesulfonic acid (HEPES) buffer (20 mM, 1 mM EGTA, 210 mM mannitol, and 70 mM sucrose, pH 7.2). SOD activity was measured according to the principle of SOD inhibition on tetrazolium salt oxidation into formazan promoted by superoxide radical. The absorbance of the formazan dye was read at 405 nm using ELISA reader (Tecan GENios, A-5082). SOD activity was expressed as unit per mg protein. For the rest of enzyme activity measurements, muscle sample was homogenized in 100 mM Tris-HCl (pH 7.5). The homogenization was centrifuged at 10,000 *g*, 4 °C for 15 min. CAT activity was measured by adding the hydrogen peroxide (H_2_O_2_) to the supernatant and the absorbance was read at 540 nm in an ELISA reader (Tecan GENios, A-5082). CAT activity was expressed as nanomole of formaldehyde per min per mg of protein. Both GPx and GR activities were measured in accordance with the protocol supplied by the manufacturer. The decrease in the absorbance of the oxidation of nicotinamide adenine dinucleotide phosphate (NADPH) can be measured at 340 nm once every minute to obtain at least 5 time points using a plate reader (Tecan GENios, A-5082). Enzyme activities were calculated per mg protein. GST activity was measured by the conjugation of GSH with 1-chloro-2,4-dinitrobenzene (CDNB). The absorbance of GSH-CDNB conjugation was read at 340 nm using an ELISA reader (Tecan GENios, A-5082). GST activity was expressed as nanomole of CDNB per minute per mg protein.

### 2.9. Xanthine Oxidase

Xanthine oxidase (XO) activity was measured based on the H_2_O_2_ production during hypoxanthine oxidation. The H_2_O_2_ reacts with ADPH (10-acetyl-3,7-dihydroxyphenoxazine) in presence of HRP (Horseradish peroxidase) to produce resourfin with high fluorescence, which was read at 535 nm (Excitation) and 585 nm (Emission) using ELISA reader (Tecan GENios, A-5082). XO activity was expressed as minute per mg of protein.

### 2.10. Citrate Synthase

CS activity was assessed using the citrate synthase kit (Sigma-Aldrich, St. Louis, MO, USA). CS activity in muscle was expressed as micromole per min per μg of protein.

### 2.11. Statistical Analysis

Two-way analysis of variance (ANOVA) and Duncan *post hoc* test were used to determine the statistical significance of mean among groups for all variables. Probability of type I error is set at 5% or less for significance. All results were expressed as mean ± standard deviation (SD).

## 3. Results

**Figure 2:**
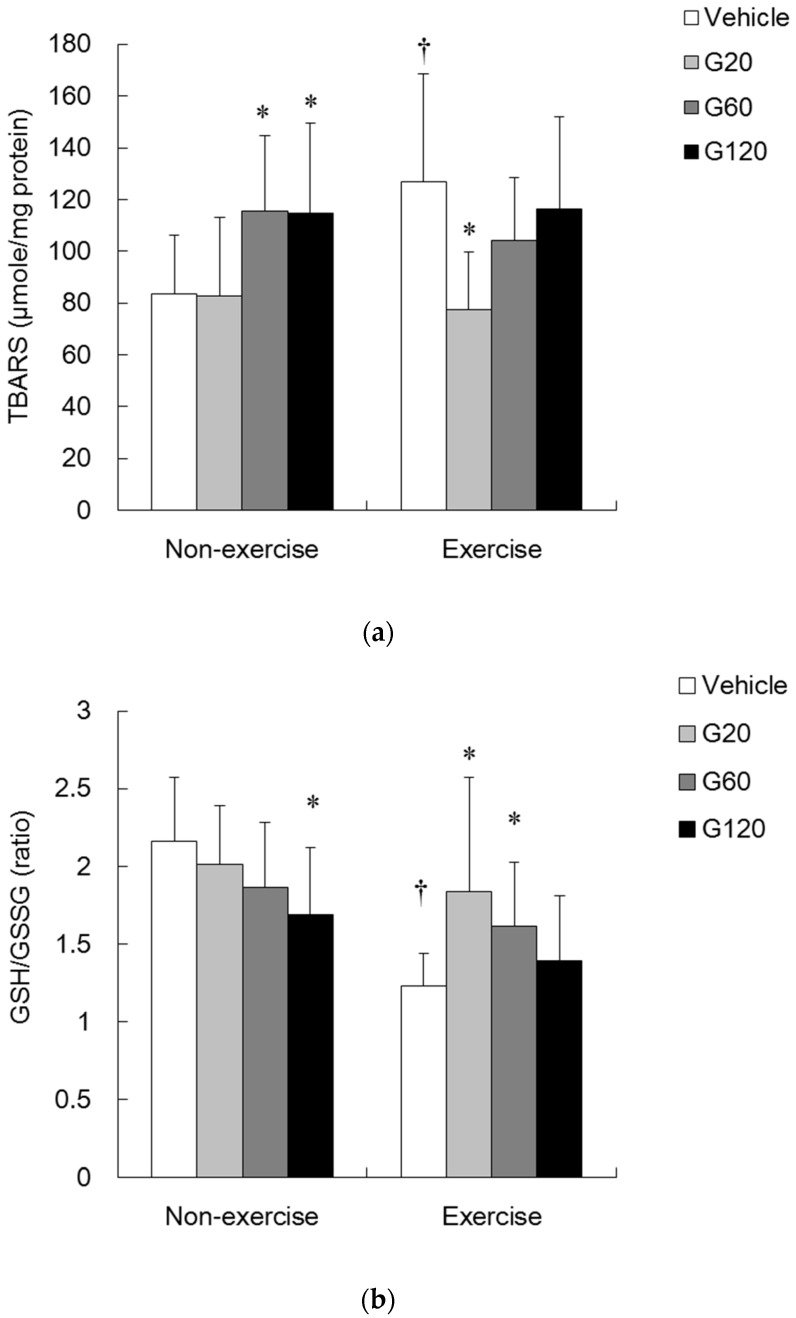
Effects of ginseng steroids on oxidative stress makers: Thiobarbituric acid reactive substances (TBARS) levels (**a**); The ratio of reduced glutathione (GSH) to oxidized glutathione (GSSG) (GSH/GSSG) (**b**) in tibialis anterior (TA) muscle. Bars are means ± SD. 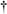 denotes significant difference against non-exercise vehicle (*p* < 0.05). 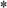 denotes significant difference against vehicle (*p* < 0.05). Vehicle: vehicle-treated in non-exercise (Non-exercise vehicle) or in exercise (Exercise vehicle) group which only received 0.9% saline for 10 weeks. G20: received 20 mg/kg body weight of ginseng steroids for 10 weeks. G60: received 60 mg/kg body weight of ginseng steroids for 10 weeks. G120: received 120 mg/kg body weight of ginseng steroids for 10 weeks.

Figure 2a shows TBARS levels of TA muscle after 10 weeks of ginseng steroid supplementation. In non-exercise rats, TBARS levels of the muscle in the G60 (60 mg/kg) and G120 (120 mg/kg) groups were significantly greater than those in the vehicle-treated group (*p* < 0.05). Exercise increased TBARS level by 50% above the non-exercise vehicle-treated group (*p* < 0.05). However, low dose of ginseng steroid supplementation (20 mg/kg) completely eliminated this increase induced by exercise. As dose increased, this protective effect of ginseng steroids was found to be diminished.

Figure 2b shows GSH/GSSG ratio of skeletal muscle after chronic ginseng steroid supplementation. In non-exercise rats, ginseng steroid decreased the GSH/GSSG ratio in a dose-dependent manner. We found a drastic decrease of GSH/GSSG ratio in tibialis anterior (TA) muscle following acute bout of exercise (*p* < 0.05). This decrease was 43% when compared with non-exercise vehicle-treated group. However, decreased GSH/GSSG ratio was attenuated by ginseng steroid supplementation at low and medium doses (20 and 60 mg/kg).

**Figure 3:**
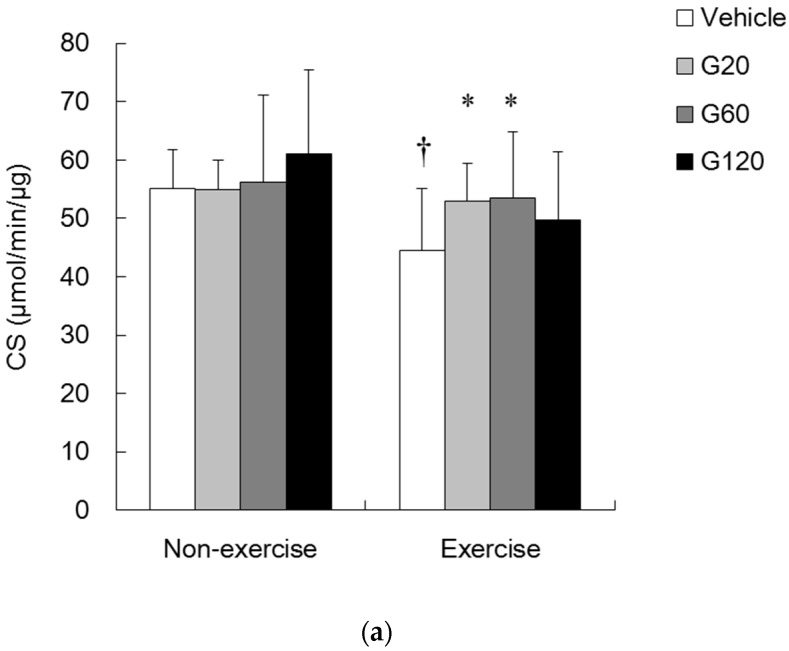

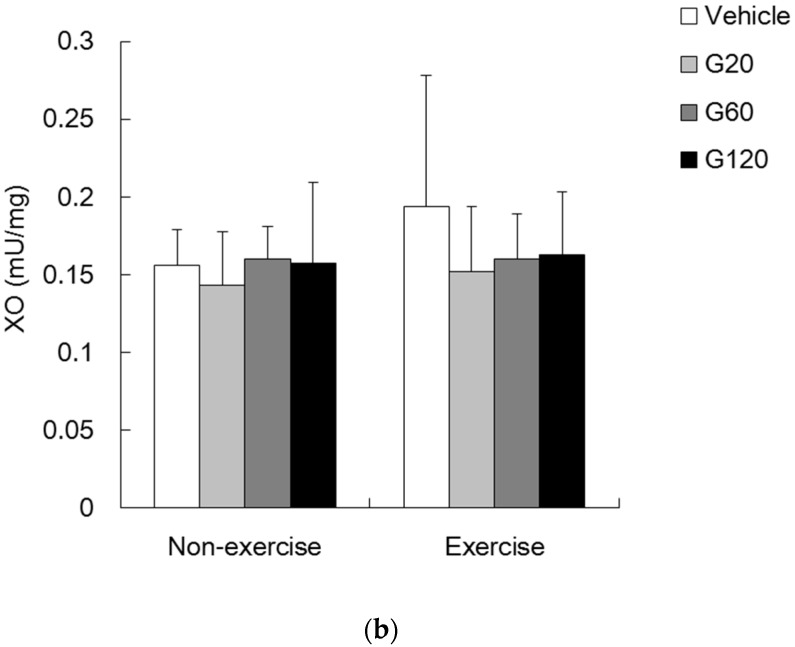
Effects of ginseng steroids on citrate synthase (CS) (**a**) and xanthine oxidase (XO) (**b**) activities in tibialis anterior muscle. Bars are means ± SD. No significant effects of exercise and ginseng steroid supplementation on muscle XO activity were found. 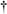 denotes significant difference against non-exercise vehicle (*p* < 0.05). 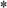 denotes significant difference against vehicle (*p* < 0.05). Vehicle: vehicle-treated in non-exercise (Non-exercise vehicle) or in exercise (Exercise vehicle) group which only received 0.9% saline for 10 weeks. G20: received 20 mg/kg body weight of ginseng steroids for 10 weeks. G60: received 60 mg/kg body weight of ginseng steroids for 10 weeks. G120: received 120 mg/kg body weight of ginseng steroids for 10 weeks.

Figure 3a presents the CS activity (a biomarker of mitochondria) of skeletal muscle. Ginseng steroid supplementation alone had no effect on muscle CS activity in the non-exercise rats. Nevertheless, muscle CS activity was significantly decreased after exercise in vehicle-treated group (*p* < 0.05), and this decrease was substantially restored by ginseng steroid supplementation at the doses of 20 mg/kg and 60 mg/kg.

**Figure 4:**
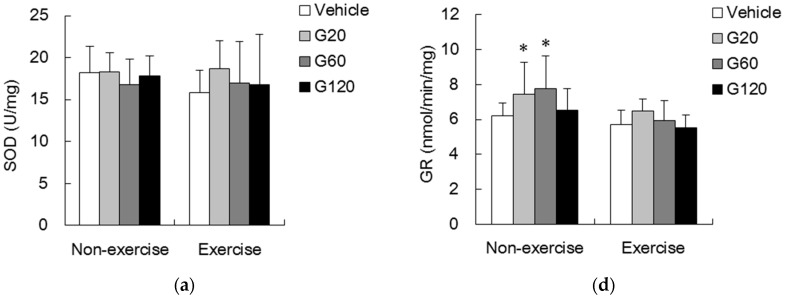

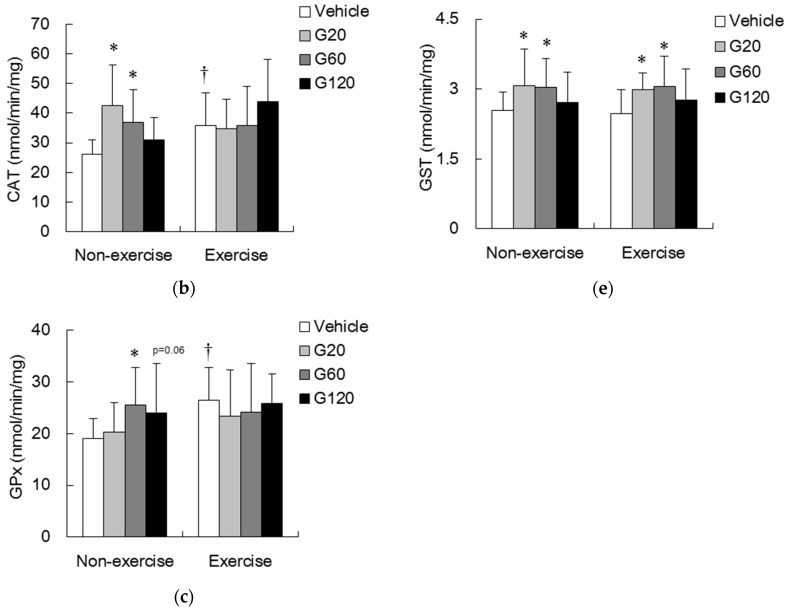
Effects of ginseng steroids on anti-oxidant enzymes: Superoxide dismutase (SOD) (**a**); Catalase (CAT) (**b**); Glutathione peroxidase (GPx) (**c**); Glutathione reductase (GR) (**d**) and Glutathione S-transferase (GST) (**e**) activities in tibialis anterior muscle. Bars are means ± SD. 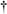 denotes significant difference against non-exercise vehicle (*p* < 0.05). 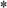 denotes significant difference against vehicle (*p* < 0.05). Vehicle: vehicle-treated in non-exercise (Non-exercise vehicle) or in exercise (Exercise vehicle) group which only received 0.9% saline for 10 weeks. G20: received 20 mg/kg body weight of ginseng steroids for 10 weeks. G60: received 60 mg/kg body weight of ginseng steroids for 10 weeks. G120: received 120 mg/kg body weight of ginseng steroids for 10 weeks.

To demonstrate the effects of ginseng steroids on free radical production and elimination, next we measured the XO and SOD activities. We found no significant effects of either ginseng steroid supplementation or exercise challenge on both XO (Figure 3b) and SOD (Figure 4a) activities in rat TA muscle.

CAT activity of skeletal muscle was shown in Figure 4b. In non-exercise rats, muscle CAT activities of the G20 and G60 groups were significantly greater than those of non-exercise vehicle-treated rats (*p* < 0.05). However, this effect was not observed with the high dose of ginseng steroid supplementation. In the vehicle-treated rats, exercise challenge increased the CAT activity by 39% above the non-exercise vehicle-treated level (*p* < 0.05). No additive effect of ginseng steroid supplementation or exercise on CAT activity was detected.

Enzyme activities of GPx, GR and GST of skeletal muscle were shown in Figure 4c–e, respectively. In non-exercise rats, muscle GPx activity was significantly higher with 60 mg/kg of ginseng steroid supplementation compared with vehicle-treated group (*p* < 0.05) (Figure 4c). Exercise challenge alone slightly increased the GPx activity by 26% above the non-exercise vehicle-treated level (*p* < 0.05). GR activities in both G20 and G60 groups were significantly greater than those in vehicle-treated group (*p* < 0.05). No exercise effect was found on muscle GR activity. For both non-exercise and exercise rats, GST activities in G20 and G60 groups were significantly higher than that in the vehicle-treated group (*p* < 0.05). 

In this study, we found no noticeable difference in swim time to exhaustion among the groups (Vehicle: 110 ± 43 min, G20: 108 ± 83 min, G60: 99 ± 55 min and G120: 105 ± 56 min).

## 4. Discussion

Previous studies reported mixed results regarding anti-oxidant status of ginseng [8,9,10]. The inconsistent metabolic actions of ginseng steroids are possibly associated with the variance in ginsenosides profile [11,16]. To avoid such discrepancy, we used standardized ginseng steroids in this study and evaluated its effects on oxidative stress and anti-oxidant status in rat skeletal muscle against an acute bout of exhaustive exercise. The major findings of the study are: (1) At rest, oxidative stress markers, TBARS was increased and GSH/GSSG ratio was decreased after a 10-week ginseng steroid supplementation in a dose-dependent manner; (2) The activities of anti-oxidant enzymes, including CAT, GR and GST were significantly increased with low and medium doses of ginseng steroids supplementation, but reversed with high dose; (3) Exhaustive exercise-induced oxidative stress was effectively buffered by low dose of ginseng steroids, as evidenced by suppressed TBARS and restored GSH/GSSG ratios. However, this buffering action was diminished with high dose of ginseng steroids; (4) The decreased mitochondrial marker enzyme, CS activity following exhaustive exercise was restored by low and medium doses of ginseng steroid supplementation. Taken together, the result of the study suggests a hormetic property of ginseng steroids on anti-oxidant status of skeletal muscle against exercise challenge.

Exhaustive exercise often causes oxidative stress [17] that is mirrored by elevated TBARS and decreased GSH/GSSG ratio following exercise [18,19]. In the present study, exhaustive exercise-induced drop in GSH/GSSH ratio was attenuated by low and medium doses (20 and 60 mg/kg) of ginseng steroid supplementation. However, this beneficial effect was diminished as dose increased, featuring a hormetic dose-response relationship. Muscle is a major source of ROS production during and after exercise. It has been stated that XO contributes to increase the oxidative stress in rat muscle after exhaustive exercise [20,21]. Thus, XO is considered as a source of free radical production. However, not all studies have shown the consistent results. A 60-min treadmill running at 27 meter per min (5% grade) does not appear to increase the XO activity [22]. Similarly, we found no significant change in XO activity in TA muscle after exercise in this study. Since muscle fiber recruitment during different exercise mode may produce diverse outcomes, future investigations are warranted to confirm the role of XO on ROS production under different types of exercise.

To the best of our knowledge, this is the first in vivo study that demonstrates the anti-oxidant action of ginseng steroids against exhaustive exercise challenge, particularly when doses are relatively low. Although ginseng steroid supplementation does not benefit to improve the exercise performance, it protects the muscle tissue from exhaustive exercise-induced oxidative damage. It has been shown that ginseng (*Panax ginseng Mayer*) extract prevented TBARS increase in rat skeletal muscle after downhill running or acute treadmill running [18,23]. The dose response results from our study indicate that increasing amount of ginseng supplementation may not enhance the tissue anti-oxidant status; instead it may produce detrimental effects on anti-oxidant status. Previous study has shown that increased dammarane-type sapogenins dose decreased in vitro cell viability [24], supporting the notion that high dose of ginseng supplementation may not be a right approach to strengthen the anti-oxidant status.

In this study, we demonstrated that both exercise and ginseng steroids can promote CAT and GPx activities, which participates in scavenging of hydrogen peroxide (H_2_O_2_). However, no additive effect of exercise and ginseng steroids on this anti-oxidant capacity was found. It is well-known that enhancing ROS buffering capability regarded as a valuable benefit of exercise training [25,26]. According to our current results, enhancing of CAT and GPx activities seems to have an upper limit in skeletal muscle. Thus combining of exercise plus ginseng steroids together may have been exceeded the adaptive capacity of skeletal muscle. 

In the present study, muscle anti-oxidant status was increased in ginseng steroid-treated non-exercise rats, which was indicated by the increased activities of CAT, GPx, GR, and GST. We further noticed a negative correlation between TBARS and CAT activity (Pearson correlation = −0.248, *p* < 0.05) and a positive correlation between GSH/GSSG ratio and GR activity (Pearson correlation = 0.248, *p* < 0.05). It is noteworthy that GST and GR were selectively responded to ginseng steroids supplementation but not to exercise challenge. This adaptation may contribute to the attenuation of exercise-induced increased ROS. Our data further suggest that the protective effect of ginseng extracts on oxidative stress reported in the past perhaps mediated by ginseng steroids. Most of the previous reports come from hepatic tissue [27] or cell-line [28]. Hormetic response was observed in CAT, GR and GST but not in GSH/GSSG ratio and CS activity. We do not know the underlying mechanism to demonstrate why some antioxidant enzyme activities show no difference between the vehicle-treated and the high dose of ginseng steroids. We speculate that high dose of ginseng steroid supplementation may lead to systemic oxidative stress elevation causes competition of stem cells among peripheral tissues, which may dilute stem cell localization into muscle tissue. It has been reported that antioxidant level is proportional to stem cell homing, proliferation and differentiation in local tissues [29,30].

Another important finding of the study is that high doses of ginseng steroid supplementation increases oxidative stress in skeletal muscle, as evidenced by increased TBARS level and decreased GSH/GSSG ratio. It has been documented that ROS act as regulators to stimulate the anti-oxidant capacity. In this context, hydrogen peroxide treatment reported to induce redox-sensitive signaling pathway and results in increased GPx gene expression in C2C12 (a mouse skeletal muscle cell line) cells [31]. In a contrary, anti-oxidant (Vitamin C and E) supplementation has been found to eliminate exercise training-induced adaptation in increasing GPx of human skeletal muscle [7]. Taken together, the anti-oxidative outcome of ginseng steroids against exercise is mediated by increasing oxidative stress. However, excessive ROS production exceeding the adaptive capacity of skeletal muscle may offset its beneficial effect. In addition, our result provides a reasonable support to suggest that an increased ROS in humans [10] and animals [18] after high dose of ginseng supplementation is mediated by ginseng steroids. 

CS is a mitochondrial enzyme, which plays an important role in aerobic capacity of skeletal muscle. CS activity of skeletal muscle can be increased by exercise training [32]. However, exhaustive exercise results in an immediate decrease of CS activity in skeletal muscle [33]. The decreased CS activity might be associated with oxidation of free thiols of the enzyme during exercise [3], suggested by an increased oxidative stress markers of exercised muscle in the present study. Furthermore, *Panax ginseng* extract has been reported to attenuate CS activity drop after acute bout of treadmill running (20 m/mm, 80 min) in exercised muscle [18]. Together with the current findings, this protective effect of ginseng against exercise challenge appears to be mediated by its steroid content.

In conclusions, here we demonstrated a hormetic dose-response relationship of ginseng steroids on anti-oxidant status of skeletal muscle following an acute bout of exercise challenge. Our findings suggesting that high dosage of ginseng supplementation should be avoided for optimizing its anti-oxidant outcomes. 

The major limitation of this study is that only TA muscle was used for analyses. TA consists of mostly fast twitch fiber, and therefore this result cannot be generalized to other muscle types with different muscle fiber composition. Furthermore, gene or protein expressions were not measured together with the anti-oxidant enzyme activities. The results of this study served as a pilot data to elicit more mechanistic investigations regarding the hormetic property of the major ginseng components.

## Figures and Tables

**Table 1:** Grouping and number of animals in each group.

	Vehicle	G20	G60	G120
Non-exercise	N = 10	N = 10	N = 10	N = 10
Exercise	N = 10	N = 10	N = 10	N = 10
